# LASTING IMPACTS OF ACES ON DEPRESSION/SUICIDAL THINKING IN BLACK DEMENTIA CAREGIVERS: A WITHIN-GROUP ANALYSIS

**DOI:** 10.1093/geroni/igae098.0789

**Published:** 2024-12-31

**Authors:** Wesley Browning, Mustafa Yildiz, Carolyn Pickering

**Affiliations:** The University of Texas Health Science Center Houston, Houston, Texas, United States; The University of Texas Health Houston, Houston, Texas, United States; The University of Texas Health Houston, Houston, Texas, United States

## Abstract

Family caregivers take on the caregiving role despite the quality of the pre-morbid relationship, and pre-morbid relationship quality is linked to many caregiving outcomes. As compared to White dementia family caregivers, Black caregivers often have lower rates of mental distress giving the misleading idea that they are not at risk for poor mental health outcomes. However, within-group analyses have been argued as a better design to understand variability of caregiver outcomes within racially minoritized groups. Therefore, we examined the long-term effects of childhood trauma on depression, depressive symptoms and suicidal thinking among Black dementia family caregivers. This study presents findings from a multi-time series study, in which participants (N=101) completed three waves including 21-day bursts of daily diary surveys (n=5,124). We utilized Generalized Linear Mixed Models (GLMM) to assess the influence of childhood physical neglect, emotional abuse, and physical abuse on risk of clinically significant depression, daily depressive symptom severity and daily suicidal thinking. GLMMs were conducted separately for each form of childhood trauma to avoid multicollinearity. GLMMs revealed all three forms of childhood trauma as risk factors for clinically significant depression over at each wave. Additionally, each form of childhood trauma was independently associated with increased daily depressive symptoms and suicidal thinking. Findings provide compelling evidence of the detrimental effects of childhood trauma on mental health among Black caregivers and help explain within-group variability. These findings underscore the need for targeted interventions aimed at mitigating the long-term mental health consequences of early-life adversity in caregiving contexts.

